# A molecular-genetic and imaging-genetic approach to specific comprehension difficulties in children

**DOI:** 10.1038/s41539-018-0034-9

**Published:** 2018-11-21

**Authors:** Miao Li, Jeffrey G. Malins, Mellissa M. C. DeMille, Maureen W. Lovett, Dongnhu T. Truong, Katherine Epstein, Cheryl Lacadie, Chintan Mehta, Joan Bosson-Heenan, Jeffrey R. Gruen, Jan C. Frijters, the GRaD Study Consortium, Richard Boada, Stephanie Gottwald, Dina Hill, Lisa A. Jacobson, E. Mark Mahone, Erik G. Willcutt, Maryanne Wolf

**Affiliations:** 1 Department of Curriculum and Instruction, College of Education, University of Houston, Houston, TX USA; 2 Graduate School of Education, Harvard University, Cambridge, MA USA; 3 Department of Pediatrics, Yale University School of Medicine, New Haven, CT USA; 4 Haskins Laboratories, New Haven, CT USA; 5 Neurosciences and Mental Health Program, Learning Disabilities Research Program, The Hospital for Sick Children, University of Toronto, Toronto, ON Canada; 6 Department of Diagnostic Radiology, Yale University School of Medicine, New Haven, CT USA; 7 Department of Genetics and the Investigative Medicine Program, Yale University School of Medicine, New Haven, CT USA; 8 Faculty of Social Sciences, Department of Child and Youth Studies, Brock University, St. Catharines, ON Canada; 9 University of Colorado Denver, Aurora, CO USA; 10 Tufts University, Medford, MA USA; 11 University of New Mexico, Albuquerque, NM USA; 12 Kennedy Krieger Institute and Johns Hopkins University School of Medicine, Baltimore, MD USA; 13 University of Colorado Boulder, Boulder, CO USA

## Abstract

Children with poor reading comprehension despite typical word reading skills were examined using neuropsychological, genetic, and neuroimaging data collected from the Genes, Reading and Dyslexia Study of 1432 Hispanic American and African American children. This unexpected poor comprehension was associated with profound deficits in vocabulary, when compared to children with comprehension skills consistent with their word reading. Those with specific comprehension difficulties were also more likely to have RU2Short alleles of READ1 regulatory variants of *DCDC2*, strongly associated with reading and language difficulties. Subjects with RU2Short alleles showed stronger resting state functional connectivity between the right insula/inferior frontal gyrus and the right supramarginal gyrus, even after controlling for potentially confounding variables including genetic ancestry and socioeconomic status. This multi-disciplinary approach advances the current understanding of specific reading comprehension difficulties, and suggests the need for interventions that are more appropriately tailored to the specific comprehension deficits of this group of children.

## Neuroscience: Biology helps explain reading comprehension difficulties

Biological data can help us better understand why as many as 15% of children struggle to comprehend what they read even when they are skilled at decoding individual words. An international team of researchers led by Dr. Jeffrey Gruen at Yale University School of Medicine collected behavioral, genetic, and brain imaging data from African American and Hispanic American children with specific difficulties in reading comprehension. These children were found to (1) be more likely to have certain variants of the reading disability risk gene *DCDC2*, (2) have stronger functional brain connectivity between the right insula/inferior frontal gyrus and the right supramarginal gyrus, and (3) have profound deficits in vocabulary. These multidisciplinary findings deepen our understanding of specific reading comprehension difficulties and pave the way for more effective reading interventions that are tailored to students with these challenges.

## Introduction

Between 7 and 15% of school-age children have normal or adequate individual word reading or decoding skills, yet also have poor reading comprehension.^1–4^ The Simple View of Reading Model suggests that successful reading comprehension requires both adequate decoding and language comprehension.^5^ Even if children have normal decoding skills, they may have poor reading comprehension if they are deficient in language comprehension.^6–8^ Recent work has shown that students with this condition, known as unexpected poor comprehension, have weak morphological processing and vocabulary skills.^1–3,6,8–12^ They can also have difficulty with higher-level cognition and language skills such as inferences, strategies, working memory, comprehension monitoring, and integration of text information.^13–15^ Early identification is key for implementing effective intervention prior to significant learning delays, but since they demonstrate adequate individual word reading or decoding, students with unexpected poor comprehension often do not receive special services.

The two essential components of reading comprehension are decoding (i.e., word reading) and language comprehension. The latter relies on a network of brain regions including inferior frontal, temporal, and inferior parietal regions.^16,17^ Many of these structures have also been associated with decoding in both children and adults^18^ underscoring the critical link between the brain networks responsible for comprehending language in its oral and written forms.^19^ However, recent work suggests that within the larger set of regions that support reading skills, the functional networks underlying decoding versus comprehension may be partially dissociated.^20,21^ In particular, both structural and functional neuroimaging studies have highlighted the contributions of the right hemisphere to processing connected discourse as opposed to processing single words.^22,23^ In addition, compared to word-level processing, discourse-level processing is more strongly associated with brain regions supporting executive functioning,^24^ which could partially account for the unexpected poor comprehension phenotype.

The functional connectivity between language and reading-related regions has been linked to variants of different genes including the dyslexia gene called *DCDC2*.^25–30^ These associations may be related to differences in neuronal migration during early development that impact the connectivity of brain networks critical for reading and language.^31^ However, imaging-genetic analyses have not yet been performed in the context of reading comprehension. We hypothesize that patterns of connectivity between brain regions associated with language comprehension will mediate potential associations between behavioral comprehension skills and genetic variants linked to reading and language.

In the current study, we tested this hypothesis by comparing the frequencies of genetic variants of a *DCDC2* expression regulator called READ1 (regulatory element associated with dyslexia (1)^32^ in children with unexpected poor comprehension, expected average comprehension, and unexpected good comprehension, from the US-Canada collaborative Genes, Reading and Dyslexia (GRaD) Study. Next we tested whether different READ1 genotypes are associated with different patterns of functional connectivity between brain regions associated with language comprehension. To do this, we collected resting state fMRI data from a subset of children in the GRaD study, and defined a brain network associated with specific comprehension skill. Finally we tested whether the strength of the connections between key nodes of the network varied as a function of READ1 genotype.

Neuropsychological measures have provided accurate assessments of poor comprehension,^6,33^ and genetics and neuroimaging have been brought together to explore disabilities in reading,^34,35^ yet how gene-brain-behavior relationships are integrated within children with unexpected poor comprehension remains unknown. Our participants were children of African and Hispanic descent. Because previous genetic studies on children with reading problems have mainly emphasized populations of European descent, with much less representation from minority groups, our study focused on representation from groups of African and Hispanic ancestry with the intention to better understand the reading comprehension problems of under-represented populations. By combining neuropsychological, genetic, and neuroimaging perspectives, we aimed to develop a comprehensive understanding of children with reading comprehension difficulties, and thereby fill an important gap in the gene-brain-behavior framework.^36,37^

## Results

### Characterization of reading comprehension groups

Groups of subjects with unexpected poor comprehension (UPC), expected average comprehension (EAC), and unexpected good comprehension (UGC) were identified using a regression method.^6,8,33^ Reading comprehension (Standardized Reading Inventory score) was regressed onto age, performance IQ, word decoding (Woodcock-Johnson Word Attack and TOWRE Phonemic Decoding Efficiency), and word recognition (Woodcock-Johnson Letter-Word Identification, TOWRE Sight Word Efficiency, Standardized Reading Inventory–Word Recognition). Reading comprehension scores were then plotted against predicted values from the regression analysis. Subjects who scored one SD below the regression line were identified as having UPC, subjects who scored one SD above the regression line were identified as having UGC, and subjects within +/− 0.5 SDs around the regression line were considered as having EAC.

Demographic information for these three groups is presented in Table 1. There were no significant differences among the three groups. Although there was no significant age difference between subjects with UPC and UGC, subjects with EAC were slightly younger than the other two groups of children. Mothers of subjects with UGC tended to have higher education, and a lower rate of social assistance, suggesting a developmental advantage in oral language that supported their higher comprehension skills.

**Table 1:** Demographic information for the three comprehension groups

	UPC (*n* = 216)	EAC (*n* = 530)	UGC (*n* = 223)
Age (M/SD)	143.75/25.12	131.19/24.30	148.17/23.59
Sex (M)	51.6%	55%	55.1%
AA^a^	41.9%	34.8%	36.9%
HA^b^	64.5%	68.3%	65.6%
Years of maternal education (M/SD)	13.30/2.51	13.24/2.94	14.44/3.02
SES^c^	59.9%	53.5%	33.8%

*Note*: *p* > 0.05 for all six variables in this table

^a^African American ancestry assignment based on self-report by the caregiver

^b^Hispanic American ancestry assignment based on self-report by the caregiver

^c^Socioeconomic status defined as percent in need of subsidy. SES was coded as a binary variable, and was determined by participation in one or more government assistance programs that have income eligibility requirements: Women, Infants, and Children (WIC), Medicaid/Husky, food stamps, or Section 8 Housing Choice Voucher

UPC: subjects with unexpected poor comprehension

EAC: subjects with expected average comprehension

UGC: subjects with unexpected good comprehension

The three groups were also compared on reading and reading-related skills (Table 2). No significant differences across groups were observed on non-verbal intelligence (WISC Matrix Reasoning), suggesting that global cognitive ability did not define the groups. Subjects with UPC met developmental expectations for word reading accuracy (WJ-III composite) and reading fluency (TOWRE composite). Both UPC and UGC groups had higher scores on these measures than the EAC group. However, in vocabulary, there were significant differences among the three groups, with UPC subjects scoring the lowest: almost one standard deviation below developmental expectations (PPVT and WISC Vocabulary). Subjects with UPC performed the worst on reading comprehension.

**Table 2:** Performance on reading and reading-related measures

Measure	Assessment	UPC (*n* = 216)		EAC (*n* = 530)		UGC (*n* = 223)		Pairwise comparisons
		M	SD	M	SD	M	SD	
Age	--	143.8	25.1	131.2	24.3	148.2	23.6	^a^UPC = UGC > EAC
PIQ	WISC	9.3	2.9	9.3	2.7	9.3	2.9	ns
Word reading accuracy	WJ-III	96.6	10.4	92.1	13.5	98.7	13.6	^a^UPC = UGC > EAC
Word reading fluency	TOWRE	94.8	14.1	90.1	16.9	96.8	16.8	^a^UPC = UGC > EAC
Reading comp	SRI	3.6	2.6	7.0	2.9	11.8	3.3	^a^UPC < EAC < UGC
Receptive vocab	PPVT	87.2	12.7	93.0	13.9	105.0	16.2	^a^UPC < EAC < UGC
	WISC Vocab	7.1	2.3	8.4	2.6	11.2	2.9	^a^UPC < EAC < UGC
Phono awareness	CTOPP	91.6	14.4	90.8	13.6	98.5	13.8	^a^UPC = EAC < UGC
Rapid auto naming	RAN	304.4	29.8	290.8	38.6	312.0	38.4	^a^EAC < UPC < UGC

*PIQ* performance IQ, *WISC* Wechsler Intelligence Scale for Children Matrix Reasoning, *WJ-III* Woodcock-Johnson III Tests of Achievement Letter Word Identification and Word Attack composite, *TOWRE* Test of Word Reading Efficiency Sight Word Efficiency and Phonemic Decoding Efficiency composite; *SRI* Standardized Reading Inventory passage comprehension, *PPVT* Peabody Picture Vocabulary Test; *CTOPP* Comprehensive Test of Phonological Processing phoneme deletion, *RAN* Rapid Automatized Naming composite

^a^ *p* < 0.05

UPC: subjects with unexpected poor comprehension

EAC: subjects with expected average comprehension

UGC: subjects with unexpected good comprehension

### Genetic analysis

READ1 genotypes – RU1-1, RU2Long, and RU2Short (Table 3) – were evaluated against the three comprehension groups. The proportion of the RU2Short genotype significantly varied across the three groups: 0.47 for UPC, 0.37 for EAC, and 0.32 for UGC, *χ*^2^(2) = 10.30, *p* *=* 0.006.

**Table 3:** Proportion of RU2Short genotype

	RU2Short genotype^a^
UPC (*n* = 216)	0.47
EAC (*n* = 530)	0.37
UGC (*n* = 223)	0.32

*Note*: Overall *χ*^2^ = 10.30, *p* *=* 0.006

^a^Proportion of subjects with at least one RU2Short genotype

UPC: subjects with unexpected poor comprehension

EAC: subjects with expected average comprehension

UGC: subjects with unexpected good comprehension

Next, to evaluate these raw proportions within the context of the present sample and important covariates, we conducted a multinomial logistic regression. The sample size permitted the inclusion of several key covariates to ensure that the results described above were robust: age, vocabulary, sex, highest level of maternal education, presence of Spanish as the primary language at home, bilingual status of the child (as determined by an interview with the primary caregiver), and socioeconomic status of the family, as well as the first 10 principal components from a set of population stratification control variables. A small amount of missing data (1.5% distributed across all datapoints) were accounted for by multiple imputation with 15 replications. Relative efficiency for predictors with missing data ranged from 0.993 to 0.999.

The overall model accounted for 19.7% of the variance (Nagelkerke pseudo *R*^2^) in predicting comprehension group membership. UPC were set as the reference group, producing two sets of coefficients: 1) prediction of UPC relative to EAC; 2) prediction of UPC relative to UGC (Table 4). Subjects in the EAC group had greater vocabulary skills (OR = 1.65; 95% CI [1.33 2.04]) and were younger (OR = 0.52; 95% CI [0.43 0.63]), compared to the UPC group. Likewise, subjects in the UGC group also had greater vocabulary skills (OR = 5.28; 95% CI [3.79 7.36]) and were younger (OR = 0.50; CI [0.39 0.66]) than the UPC group. In contrast, low SES was less likely in UGC (OR = 0.58; CI [0.36 0.93]) compared to UPC, as was having a mother with higher education (OR = 0.87; CI [0.031 0.66]). Controlling for all these factors, our target variable – the presence of READ1 RU2Short alleles – was also a significant predictor of having poor comprehension skills despite adequate word reading (OR = 0.6; 95% CI [0.38 0.95]).

**Table 4:** Results from the multinomial logistic regression analysis identifying predictors of EAC or UGC, relative to UPC

Group	Predictor	OR	*p*	95% CI for OR	
				Lower	Upper
EAC vs. UPC	Age	0.52	<.001	0.43	0.63
	Sex (1 = Female)	1.12	.497	0.82	1.53
	Receptive vocabulary	1.65	<.001	1.33	2.04
	Socioeconomic status (1 = Low)	0.75	.748	0.53	1.07
	Spanish primary language in home	0.64	.073	0.40	1.04
	Bilingual status (1 = bilingual child)	1.22	.399	0.77	1.93
	Highest level of maternal education	0.99	.921	0.81	1.21
	READ1 RU2Short (1 = Present)	1.23	.232	0.88	1.73
UGC vs. UPC	Age	0.50	<.001	0.39	0.66
	Sex (1 = Female)	1.13	.553	0.75	1.72
	Receptive vocabulary	5.28	<.001	3.79	7.36
	Socioeconomic status (1 = Low)	0.58	.024	0.36	0.93
	Spanish primary language in home	0.58	.120	0.29	1.15
	Bilingual status (1 = bilingual child)	1.07	.838	0.55	2.10
	Highest level of maternal education	0.87	.031	0.66	1.14
	READ1 RU2Short (1 = Present)	0.60	.030	0.38	0.95

UPC: subjects with unexpected poor comprehension

EAC: subjects with expected average comprehension

UGC: subjects with unexpected good comprehension

We performed three sensitivity analyses to ensure that our results were not due to the analytic model strategy. First, classification of comprehension groups was reconfigured to include participants between 0.5 and 1.0 SD on the residualized comprehension score. The same multinomial logistic regression was performed with the three groups, with identical results. Second, raw residualized comprehension scores (representing the degree to which the subject had good/poor reading comprehension relative to decoding) were regressed onto the same predictors in a linear regression model. This model showed the same effect for READ1 RU2Short (*p* = .01). Third, to address potential concerns that variation in English-language proficiency may have influenced the results, we repeated the logistic regression excluding bilingual students in one analysis, and participants in which Spanish was the primary language spoken in the home in the next. In both cases, the focal effect for READ1 RU2Short was preserved, and slightly increased in strength (*p* = .002 and *p* = .003, respectively).

### Neuroimaging analyses

**Fig. 1:**
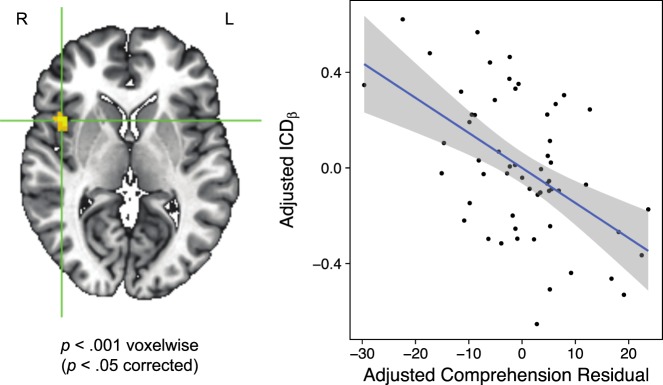
The region in right insula/inferior frontal gyrus that showed a significant association between comprehension residuals and ICD beta parameters

We analyzed resting state functional connectivity in a subset of 58 children from the larger GRaD sample, 20 with at least one copy of the RU2Short genotype. For this analysis, comprehension skill was treated as a continuous variable to maximize statistical power, and was defined by residualizing comprehension performance on age and raw scores for performance IQ, word reading, and decoding. We first performed a groupwise analysis of beta parameters characterizing the intrinsic connectivity distribution (ICD).^38^ ICD beta parameters index the scale of the distribution of correlation strengths between each voxel and all other voxels in the brain. This analysis isolated a region in right insula/inferior frontal gyrus (located at 44, 8, 6 in Talairach space; Fig. 1) that showed an association between comprehension residuals and ICD beta parameters, even when controlling for sex, SES, maternal education, and the first three genetic ancestry principal components (PCs). As shown in Fig. 1, the direction of the relationship between comprehension residuals and ICD beta parameters was negative. That is, ICD beta values were larger for children with negative comprehension residuals (analogous to UPC) than they were for children with positive comprehension residuals. This result indicates a greater density of strong correlations in the right hemisphere in subjects with UPC compared to UGC. Examining Fig. 1, there is the possibility that a few extreme data points may be influencing the model. To ensure this was not the case, we first re-evaluated the partial correlation while restricting the range of comprehension residuals to values between +/− 20, and found that the relationship between comprehension residuals and ICD beta values was still significant (*p* = .0013). Second, we quantified Cook’s distance as well as DFBETA for each data point and found that three points passed the threshold to be considered influential observations. We then repeated the analysis without these three data points and the significant focal relationship still remained (*p* = .00015).

**Fig. 2:**
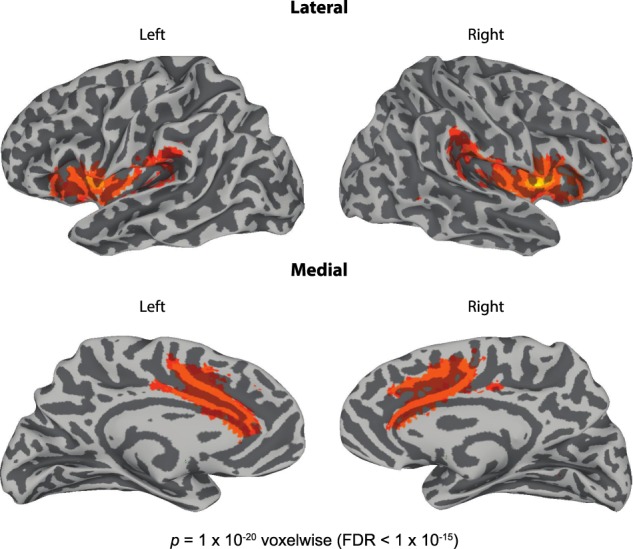
The network of brain regions whose resting state time courses correlated with the seed region in the right insula/inferior frontal gyrus. This network included left insula/IFG, right and left supramarginal gyrus, and the anterior cingulate cortex

Using the cluster in the right insula/inferior frontal gyrus (IFG) defined from the ICD analysis, we then defined a resting state network of regions with time courses that were correlated with the seed time course. As shown in Fig. 2, across subjects the right insula/IFG was highly correlated with its homologous region in the left hemisphere (peak voxel located at −41, 5, 6) as well as bilateral supramarginal gyrus (SMG; peak voxel for the left located at −56, −26, 18, and peak voxel for the right located at 56, −29, 21) and the anterior cingulate cortex (ACC; peak voxel located at 5, 17, 30).

**Fig. 3:**
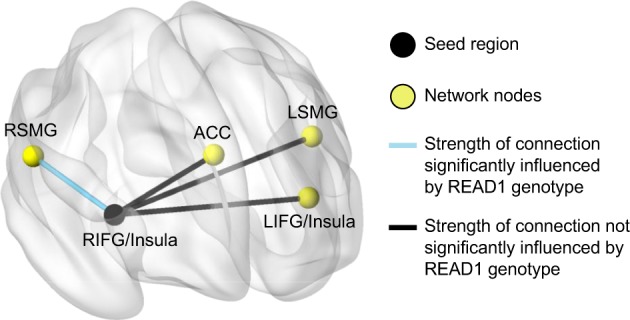
The functional connection from the right IFG seed region to the right SMG that was stronger in the RU2Short group compared to the non-RU2Short group. Network visualization was performed using BrainNet Viewer.^71^ RIFG right inferior frontal gyrus, RSMG right supramarginal gyrus, LIFG left inferior frontal gyrus, LSMG left supramarginal gyrus, ACC anterior cingulate cortex

Next, we used multiple regression analysis to assess the relationship between READ1 genotypes and the strength of the functional association between the right insula/IFG seed and fellow nodes in the resting state network indentified by the seed-based connectivity analysis. We first used backward selection to identify important covariates to include in final models. The initial pool of covariates included age, sex, performance IQ, SES, maternal education, and the first three genotype PCs. Based on these analyses, sex and/or SES were included as covariates in the final models. As shown in Table 5 and illustrated in Fig. 3, READ1 genotypes were significantly associated with the strength of the functional association between the right insula/IFG seed and the right SMG. That is, functional connectivity between the right insula/IFG and the right SMG was stronger in subjects with RU2Short alleles than it was in subjects without RU2Short alleles. It should be noted that for three of the four connections tested (left insula/IFG, left SMG, and right SMG), SES accounted for significant variance in functional connectivity, whereas in the ACC, none of the regressors accounted for significant variance in functional connectivity (and so the ACC is not listed in Table 5).

**Table 5:** Results from the multiple regression analysis characterizing the association between READ1 genotypes, as well as alternative regressors, on the strength of the functional association between the right insula/IFG seed and fellow nodes in the resting state language comprehension network

Regressor	Left insula/IFG			Left SMG			Right SMG		
	*B*	*SE*	*p*	*B*	*SE*	*p*	*B*	*SE*	*p*
READ1 RU2Short (1 = Present)	.065	.049	.189	.063	.040	.117	.077	.037	.042
Sex (1 = Female)	–	–	–	–	–	–	.082	.035	.022
SES (1 = Low)	.143	.059	.019	.132	.048	.008	.103	.045	.025

*Note*: SES was coded as a binary variable, and was determined by participation in one or more government assistance programs that have income eligibility requirements (Women, Infants, and Children [WIC]; Medicaid/Husky; food stamps; and Section 8 Housing Choice Voucher Program, Section 8)

*IFG* inferior frontal gyrus, *SMG* supramarginal gyrus

## Discussion

As children move out of the primary grades, they transition from “learning to read” to “reading to learn”. Mastering the content of the elementary and higher grades places a high demand on reading comprehension. In this study, we used neuropsychological, genetic, and neuroimaging methods to characterize specific reading comprehension difficulties among a group of Hispanic American and African American children.

Broad consensus is lacking on how to define specific reading comprehension difficulties.^4,39^ Some studies have used a sample-specific rank order definition.^4,12^ For example, this method would define UPC by scores below the 20th–30th percentile on reading comprehension but above the 40th–50th percentile on word reading or decoding skills. In the present study, we regressed reading comprehension onto word reading and/or decoding scores, defining UPC as one SD below the regression line.^2,8,33^ We also adjusted for age and performance IQ in the regression that defined the groups. The regression method accounts for the correlation between reading comprehension and word reading skill to establish more reliable estimates of specific comprehension difficulties.

The sample of subjects with UPC in our study had average to above average word-level reading skills, but performed poorly on reading comprehension, on average one to two standard deviations below developmental expectations. This group is well accommodated within the Simple View of Reading model, in which poor comprehension can be driven by poor language comprehension rather than by decoding.^5^ Subjects with UPC were somewhat behind developmental expectations on phonological awareness. The strongest deficit found within the UPC group was in vocabulary. Consistent with previous studies, this suggests that the poor performance in reading comprehension may be attributed to difficulties in vocabulary and semantic processing.^1,2,6,9^

### The gene-behavior link

The familial nature of reading performance and dyslexia has been well documented and formally studied through segregation analyses in both large pedigrees as well as nuclear families.^40–42^ Associations with specific genetic variants predicated on a range of reading skill assessments, endophenotypes, and composite scores – some that have been replicated in different languages – are well described.^43,44^ While the heritability of UPC is unknown, the heritability of specific reading skills including comprehension, as well as dyslexia, ranges from 54 to 85%.^45–48^ Of the nine described dyslexia loci (DYX1- DYX9), the DYX2 locus, located on chromosome 6p22, is the most replicated and contains the dyslexia risk gene called *DCDC2*.^27–29^ READ1 (regulatory element associated with dyslexia 1), a highly polymorphic complex repeat with at least 40 alleles,^28^ is a regulatory element that modulates expression of *DCDC2* and is encoded in the second intron.^28,30,31^ Clinical studies show allele-specific association between READ1 and word-level reading, as well as severe language impairment,^36^ making for an ideal candidate for testing association with comprehension.

In the genetic analysis, there was a high percentage of subjects in the UPC group that had at least one copy of READ1 RU2Short alleles. This was prominent in the UPC/UGC contrast, suggesting that the READ1 effect is strongest when discrepancy between decoding and higher-level skills is largest. In particular, a limitation of past studies on specific comprehension deficits is that they have collapsed EAC and UGC groups.^11,12^ As a result, past significant findings may have been driven by subjects with UGC.

An important limitation of the sample is noted. While every effort was made to only enroll and test children with sufficient English-language proficiency to validly complete the assessments, there is the possibility that the varying language status within the Hispanic participants may have influenced our results. As evidence that this is not likely a major concern, when the primary analysis was repeated without bilinguals and/or those who lived in a Spanish language home context, the effect of READ1 on unexpected comprehension difficulties was preserved. This suggests that the effects demonstrated here may be generalizable to a mixed English-language proficiency population. However, caution is still warranted as we did not have a large enough sample to investigate language as a potential moderator of these effects.

### The gene-brain-behavior link

Upon establishing a genetic-behavior link between reading comprehension and READ1, we assessed how functional connectivity mediates this relationship. By examining resting state functional connectivity in 58 children from the larger GRaD sample, we discovered that children with RU2Short READ1 alleles exhibited greater right hemisphere connectivity compared to children without RU2Short alleles. It should be noted that there were only 58 participants in the imaging-genetic analyses and we treated the comprehension residual phenotype as a continuous variable in order to maximize power. While this differs from the categorical treatment of comprehension residuals in the behavior-genetic analysis, it is unlikely to have had a significant effect on the results.

Using the comprehension residual phenotype, we identified a seed region in the right insula/inferior frontal gyrus (IFG) that showed a negative relationship between comprehension residuals and intrinsic connectivity measures. This association with the right IFG is consistent with previous studies linking the structure and function of right prefrontal regions to reading comprehension in typically developing readers,^22,23^ as well as a study demonstrating that gray matter thickness in right prefrontal regions can be used to differentiate adolescents with specific reading comprehension deficits from typically developing readers and individuals with developmental dyslexia.^49^ Using this right insula/IFG seed region, we identified a resting state network of brain regions including bilateral insula/inferior frontal gyri, bilateral supramarginal gyri (SMG), and the anterior cingulate cortex (ACC). Bilateral IFG and bilateral SMG comprise part of a resting state network associated with language comprehension,^16,17^ whereas bilateral insula and the anterior cingulate are more closely associated with the salience network.^50^ It is possible that the right SMG region we observed may also be a component of the fronto-parietal attention network, which may play a stronger role in discourse-level processing compared to processing single words.^24^

Beyond just validating the genetic link between *DCDC2* and brain regions associated with reading comprehension, our results show that RU2Short is associated with the strength of the functional association between the right insula/inferior frontal gyrus and the right supramarginal gyrus. Therefore, patterns of connectivity in the right hemisphere may be useful in interpreting the behavior-genetic relationships that we observed. Stronger connectivity between right prefrontal and posterior structures has been previously documented in poor readers compared to typically developing readers,^51^ and is thought to reflect atypicality in the lateralization of the reading network, which is left hemisphere dominant in skilled readers.^52^ However, the current study is the first time that patterns of right hemisphere connectivity have been reported in the context of poor reading comprehension within a gene-brain-behavior framework. While further research is required to validate this link, we propose that an individual’s READ1 genotype could be a useful marker for the organization of the functional circuitry for language in the brain.

READ1 genotype appeared to contribute to unique variance in functional connectivity beyond other factors previously associated with differences in connectivity, including sex and SES. However, we note that SES accounted for significant differences in connectivity in three of the four network connections that we tested. The direction of the effect was such that greater right hemisphere connectivity was observed in children from low compared to high SES backgrounds. This finding complements previous associations between SES and differences in brain structure^53^ and neurocognitive functioning,^54^ as well as the observation that brain-behavior relationships critical for reading development are more pronounced in low SES environments.^55^

Our results extend previous studies demonstrating that *DCDC2* is associated with word-level reading difficulties or language impairment,^28–30,32,56,57^ but suggest that READ1 influences reading comprehension and/or higher-level skills independent of the associations with word-level reading. Reading comprehension is a complex trait and is influenced by many genetic variants of small effect size.^58^ READ1 alleles are not the sole contributing factor to variation in reading comprehension, and using READ1 alleles in isolation will not identify children who will develop poor comprehension or those who will benefit from reading interventions. However, READ1 is still important to study in the context of reading comprehension and dyslexia, in general, because luciferase reporter studies have shown that READ1 plays a role in the regulation of *DCDC2* expression, which is one of the most replicated genes for dyslexia.^30–32^ Furthermore, animal models with a genetic knockout of *DCDC2* show impaired working memory, which has also been observed in poor reading comprehension.^59^ Although READ1, on its own, is not a suitable target to identify children with poor reading comprehension and beneficiaries of reading interventions, our findings, which show a relationship between RU2Short and right insula/IFG and the right SMG, provide initial insights into neurobiological mechanisms that we can target for future studies to understand the etiology of poor reading comprehension within a gene-brain-behavior context.

The current study is unique because we investigated reading comprehension difficulties using three different disciplinary perspectives: neuropsychology, genetics, and neurobiology. Subjects with UPC exhibited deficits in vocabulary, were more likely to have RU2Short READ1 alleles within *DCDC2*, and also showed stronger resting state functional connectivity between the right insula/IFG and the right SMG. Our findings increase the understanding of the genetic dynamics and their link to brain functions, moving a step towards early identification of poor comprehension. This multi-disciplinary approach advances the current understanding of reading comprehension difficulties, and helps inform early and accurate identification of children at risk for developing reading comprehension difficulties prior to significant delays in later grades. Many current evidence-based reading interventions devote significant time to remediating poor decoding skills. Early identification of specific comprehension deficits affords the opportunity to implement early intervention programs targeted to comprehension deficits in the presence of adequate decoding skills.

## Methods

### Subjects

Subjects were 1,432 African American and Hispanic American children, ages 8 to 15 years. This study was part of a larger, multi-site US and Canadian collaborative Genes, Reading, and Dyslexia (GRaD) project led by Yale University (PI: JR Gruen) that followed a case:control design. Inclusion criteria for cases with RD were either history of poor reading skills, reports of skills falling below expected levels for age or grade, and/or provision of special services in the area of reading. Inclusion criteria for controls were reading skills above current expectations for grade, and performance above the 40th percentile on standardized school or clinical testing. Exclusion criteria were age outside the target age range; non-minority ethnic or racial group membership; foster care placement; preterm birth (<36 weeks gestation); prolonged stay in the NICU after birth (>5 days); history of diagnosed or suspected significant cognitive delays, significant behavioral problems, or frequent school absences; history of serious emotional/psychiatric disturbances (i.e., major depression, psychotic or pervasive developmental disorder, Autism) or a chronic neurologic condition (i.e., seizure disorder, developmental neurological conditions, Tourette’s or other tic disorders, acquired brain injuries); and documented vision or hearing impairment. Parental consent forms and child assent were collected before participation. This study was approved by the Human Investigation Committee of Yale University and all of the review boards of participating data collection sites.

### Population stratification correction

A principal components analysis on genome-wide genotyped single nucleotide polymorphisms (SNPs) was conducted to model continuous axes of genetic variation within the GRaD sample using EIGENSTRAT.^60^ Prior to analysis, SNP level quality control included the removal of SNPs that met the following criteria: missingness greater than 5%, Hardy-Weinberg equilibrium *p* < 0.0001, not autosomal, or a minor allele frequency less than 0.05. Sample level quality control included the removal of samples with percentage of missing genotypes greater than 3% and samples with discrepancies between reported and inferred sex based on X chromosome heterozygosity. The first 10 principal components were used to correct for genomic inflation due to allele frequency differences across different ancestries. Analyses of genome-wide SNP data in the GRaD study found that the first 10 principal components were effective in reducing the effects of population stratification (genomic inflation factor <1.05) in the phenotypes evaluated in the present analysis.^61^

### Neuroimaging

Neuroimaging data was collected from 100 subjects who were randomly recruited with parents’ consent in the GRaD study. Of these, data from 58 subjects (27 female; ages 8.0 to 15.75) passed quality control procedures for resting state fMRI (i.e., having at least 60% of retained images across two runs, after censoring volumes which exceeded the thresholds of 0.3 mm point-to-point Euclidean movement and/or 10% outlier voxels; one subject was also removed from analysis because of having a dual deletion for READ1). The 42 participants who were excluded for data quality purposes were significantly younger than the included participants, and consequently had lower raw scores on Woodcock-Johnson III Letter-Word Identification and SRI Passage Comprehension. Importantly, however, the included and excluded participants did not significantly differ in age-normed standard scores for any test. The included and excluded participants also did not differ in the comprehension residual measure, nor did they differ in ancestry, SES, or in the proportion of participants with RU2Short versus non-RU2Short READ1 alleles. Therefore, we are confident that the reported results are not biased by the exclusion of participants for data quality purposes. Euclidean movement was calculated per volume as the square root of the sum of squares of point-to-point change for each of the six motion parameters (i.e., three translation and three rotation). Each child was assigned to one of two groups depending on READ1 genotype (Table 6): RU2Short, or non-RU2Short.

**Table 6:** Demographic characteristics concerning the 58 subjects (27 female) who had usable resting state fMRI data

Measure	Means of assessment	Summary		
		Mean	SD	Range
Age	–	10.8	2.0	8.0–15.75
Performance IQ	WISC Matrix Reasoning raw score (out of 35)	18.3	5.4	7–32
Word reading	TOWRE Sight Word Efficiency raw score (out of 104)	63.4	13.5	17–98
	WJ-III Letter Word Identification raw score (out of 76)	50.1	8.9	30–66
Pseudoword decoding	TOWRE Phonemic Decoding Efficiency raw score (out of 63)	30.1	13.9	1–52
	WJ-III Word Attack raw score (out of 32)	19.0	6.8	4–30
Reading comprehension	SRI passage comprehension raw score	37.9	16.7	2–87
Maternal education	Number of self-reported years	11.7	3.2	6–18
Socioeconomic status (SES)	Participation in a government assistance program^a^	47 indicated participation; 11 did not		
Genetic ancestry	Population stratification correction based on principal component analysis	First three principal components used as control variables		
READ1	–	20 RU2short; 38 non-RU2short		

SES was coded as a binary variable, and was determined by participation in one or more government assistance programs that have income eligibility requirements (Women, Infants, and Children (WIC); Medicaid/Husky; food stamps; and Section 8 Housing Choice Voucher Program, Section 8)

Demographic characteristics for the 58 subjects with usable resting state fMRI data are presented in Table 6. For each subject, comprehension residual values were calculated by regressing reading comprehension scores onto age, performance IQ, word decoding, and word recognition. Comprehension residuals ranged from −33 to 27 (*M* = .995; *SD* = 11.2).

### Neuropsychological measures

Reading outcome assessments were standardized measures including Woodcock-Johnson III – Letter-Word Identification and Word Attack,^62^ Test of Word Reading Efficiency – Sight Word Efficiency and Phonemic Decoding Efficiency (TOWRE),^63^ and Standardized Reading Inventory – Word Recognition and Reading Comprehension (SRI).^64^ Core reading-related skill measures included well-validated instruments tapping phonological awareness assessed by the Comprehensive Test of Phonological Processing – Elision and Blending (CTOPP),^65^ naming speed assessed by the Rapid Automatized Naming – Letters, Numbers, and Objects (RAN),^66^ receptive vocabulary assessed by the Peabody Picture Vocabulary Test (PPVT)^67^ and the Wechsler Intelligence Scale for Children – Vocabulary (WISC).^68^ Performance IQ was measured by the Wechsler Intelligence Scale for Children – Matrix Reasoning (WISC).^68^

### Woodcock–Johnson Tests of Achievement, third edition (WJ-III)

Measures of interest from the WJ-III included the Letter-Word Identification and Word Attack subtests.^62^ The WJ-III Letter Word Identification subtest is an untimed measure of non-contextual single word reading ability requiring the child to read a list of increasingly complex English words aloud. The Word Attack subtest asks the subject to apply knowledge of English phonology to decode non-words or pseudowords in isolation. The total score for each subtest represents the number of words read correctly, converted to a standard score based upon age norms.

### Test of Word Reading Efficiency (TOWRE)

The TOWRE is a fluency assessment of single word reading and single pseudoword decoding under timed conditions.^63^ The subject is asked to read as many individual words (Sight Word Efficiency) or non-words (Phonemic Decoding Efficiency) of increasing length and phonetic difficulty as possible in 45 s. Scores for Sight Word Efficiency and Phonemic Decoding Efficiency represent the number of correctly read words within the time limit, relative to age norms.

### Standardized Reading Inventory, second edition (SRI)

The SRI is an individually-administered contextual reading test that consists of 10 passages of increasing difficulty, ranging from pre-primer to an eighth grade level.^64^ Oral reading accuracy is assessed and subjects are then asked to answer a series of comprehension questions. Scores are obtained for word recognition accuracy and comprehension on each passage; the total score in each skill area is converted to a norm-referenced standardized score.

### Comprehensive Test of Phonological Processing (CTOPP)

CTOPP is a comprehensive instrument designed to assess phonological awareness, phonological memory, and rapid naming.^65^ In the current study, Elision and Blending Words subtests were used to measure phonological awareness. Elision measures the ability to remove phonological segments from spoken words to form other words. Blending Words measures the ability to synthesize sounds to form words.

### Rapid Automatized Naming and Rapid Alternating Stimulus tests (RAN)

The Letters, Digits, and Objects subtests from the RAN assesses speeded lexical retrieval, requiring the rapid naming of a series of letters, digits, and objects (repeated randomly in 5 rows of 10 items) as quickly as possible without making mistakes.^66^ Time to completion is recorded and converted to an age-referenced standard score.

### Peabody Picture Vocabulary Test, fourth edition (PPVT-4)

The PPVT-4 is an untimed measure of receptive vocabulary knowledge; the subject is required to point to one of four pictures that best indicates the target word presented.^67^ Total raw scores (correct responses) are converted to age-normed standardized scores.

### Wechsler Intelligence Scale for Children, fourth edition (WISC-IV)

The WISC-IV Vocabulary measures verbal fluency, concept information, word knowledge, and word usage.^68^ Subjects are asked to give oral definitions of words. Scoring was 2-1-0, according to the quality of the responses. The WISC-IV Matrix Reasoning measures performance IQ.

### DNA collection and genotyping

DNA was collected from saliva using Oragene-DNA kits (DNA Genotek Inc.) and extracted with prepIT-L2P (DNA Genotek Inc.) using manufacturer protocols. READ1 genotyping was conducted using PCR amplification and purified of PCR reagents. Sanger sequencing was performed at the Yale W.M. Keck DNA Sequencing Facility using standard protocols. Primer sequences and amplification protocols are described in detail elsewhere.^30^ READ1 genotypes were called from chromatograms using a custom program written in C + + (Dr. Yong Kong, available upon request). If the calling program identified errors, chromatograms were manually examined and deconvoluted for genotype calls. The genotype call rate for READ1 alleles in the GRaD sample was 0.987.

Allele-specific PCR was used to genotype the 2445 bp microdeletion in intron 2 of *DCDC2*, which encompasses READ1. Primer sequences and amplification protocols for microdeletion genotyping are described in detail elsewhere.^30^ The deletion genotype call rate was 0.972 in the GRaD sample.

### Functional groups of READ1 alleles

READ1 is composed of seven repeat units; however, grouping of alleles was based on variation in the first two repeat units. READ1 alleles were assigned to one of three groups (Table 7). RU1-1 alleles have only one copy of Repeat Unit 1 (RU1-1: alleles 2, 3, 9, 12, 25, 27). RU1 sequence was previously used to capture ETV6, a dimerizing transcriptional repressor that binds to a consensus binding site, GGAAG, which is present in the RU1 site: GAGAGGAAGGAAA. The RU1-1 group of alleles was associated with a moderate protective effect on reading performance in the Avon Longitudinal Study of Parents and Children (ALSPaC), a longitudinal cohort of European descent.^32^ RU1 also contains a consensus binding site (GAGAGGAAGGAaagg) for many C2H2 domains that define classical zinc finger transcription factors.

**Table 7:** READ1 allele groups and previously reported associations

Allele group	Definition	Alleles	Phenotype
RU1-1	One copy of Repeat Unit 1	2, 3, 8, 12, 25, 27	Protective effect for RD in ALSPaC (Powers et al.^32^)
RU2Long	> = 8 copies of Repeat Unit 2	5, 6, 13, 14, 19, 20, 22, 23	Poor reading performance in ALSPaC (Powers et al.^32^)
RU2Short	< = 6 copies of Repeat Unit 2	4, 10, 15, 16, 17, 21, 24, 26, 30, 37, 39	Poor reading comprehension in GRaD

RU2Long alleles have two copies of RU1 and greater than seven copies of Repeat Unit 2 (RU2: alleles 5, 6, 13, 14, 19, 20, 22, 23; Table 7). RU2 (GGAA) also contains consensus-binding sites for ETV6. In the ALSPaC, allele 5 was associated with increased risk for RD, whereas allele 6 was associated with increased risk for LI.^32^

RU2Short is characterized by alleles that have fewer than six copies of RU2 (4, 10, 15, 16, 17, 21, 24, 26, 30, 37, or 39). The most frequent alleles in this category are alleles 4 and 10, which have not shown association with either a positive or negative impact on reading performance in our previous studies with the ALSPaC (see Table 7). Note that because of this coding scheme, children with one copy of the deletion of READ1 were essentially treated as being homozygous for the other allele.

### Magnetic resonance imaging data

Images were collected using a single 3 T TIM-Trio scanner located at the Yale Magnetic Resonance Research Center in New Haven, CT. Anatomical images were acquired in a sagittal orientation (MPRAGE; matrix size = 256 × 256; voxel size = 1 × 1 × 1 mm; FoV = 256 mm; TR = 2530 ms; TE = 2.77 ms; flip angle = 7°). T2*-weighted images were collected in an axial oblique orientation (25 slices; 6 mm slice thickness; no gap) using single-shot echo-planar imaging (matrix size = 64 × 64; voxel size = 3.44 × 3.44 × 6 mm; FoV = 220 mm; TR = 1550 ms; TE = 30 ms; flip angle = 80°). Subjects were asked to keep their eyes open to the best of their ability, but were told that they could blink if needed. Two runs of resting state fMRI were administered, each 6:18 in length (240 volumes), for a total of 12:36 (480 volumes). Within each run, the first six volumes were removed to allow for stabilization of the magnetic field.

### Preprocessing of MRI data

Resting state functional images were preprocessed in AFNI^69^ by first performing despiking (*3dDespike*) and slice-scan time correction (*3dTshift*). Next, functional images were co-registered with anatomic images, were warped to the Talairach template using a non-linear transform (*3dQWarp*), and were corrected for motion by alignment to the volume with the minimum outlier fraction (*3dvolreg*). These three steps were concatenated into a single transform that also forced a 3 mm isotropic voxel size on the data. Data were then smoothed using a 6 mm FWHM Gaussian kernel (*3dmerge*). Next, a general linear model (GLM) analysis was performed (*3dDeconvolve*) using regressors for the six motion parameters as well as their derivatives, and regressors for low frequency components less than 0.01 Hz. In this GLM, volumes were censored if they exceeded the threshold of 0.3 mm point-to-point Euclidean movement and/or 10% outliers. Following this, tissue-based regression was performed to regress out the first three principal components of each subject’s eroded lateral ventricle mask as well as the average signal in each subject’s eroded white matter mask. The latter was performed using the “fast” *ANATICOR* program in AFNI.^70^ White matter and lateral ventricle masks were created for each subject by segmenting and parcellating anatomical scans in Freesurfer^71^ using the Desikan-Killiany atlas.^72^

### Defining seeds using the intrinsic connectivity distribution

Using custom scripts written in R, which were adapted from Scheinost et al. (2012),^73^ intrinsic connectivity distribution (ICD) maps were created for each subject by assessing voxelwise connectivity in the residual maps from the GLM. For each voxel, a histogram was generated that characterized the density of correlations to every other voxel in the brain. These histograms were approximated using a stretch exponential function. Two parameters characterizing this function, alpha and beta, respectively index the scale and shape of the distribution. Smaller values of the alpha parameter and larger values of the beta parameter both indicate a larger degree metric, or more extensive connectivity, at any correlation threshold. The package *oro.nifti*^74^ was used to read and write NIfTI files. ICD values were only generated for voxels within a gray matter mask, which included cortical and subcortical structures as well as the brainstem.

To define seeds for functional connectivity analyses, we performed a whole-brain analysis on subject-wise ICD parameters. To facilitate group comparison, ICD maps were first smoothed with a 6 mm FWHM Gaussian kernel (*3dmerge*). Groupwise analysis was performed in AFNI using a multivariate model (*3dMVM*) with ICD parameters as the dependent variable and comprehension residuals (reading comprehension regressed onto age, performance IQ, word decoding, and word recognition) as the independent variable. This model also included the following covariates: sex, number of years of maternal education, SES, and the first three genotype principal components. Separate models were performed with ICD alpha and beta parameters as respective dependent variables; results reported here are for the beta parameter. For these groupwise analyses, the voxelwise threshold was *p* = .001, cluster corrected at *p* = .05. Cluster correction was performed by estimating the spatial autocorrelation of the residual time course for each subject (*3dFWHMx*), and then using mean parameter values across subjects as inputs to the simulation program *3dClustSim* (10,000 iterations). This procedure was based on the latest recommendations for cluster correction in fMRI.^75^ For this simulation, the size of the search space was constrained to the number of voxels in the gray matter mask for which ICD measures were calculated.

### Assessing the association between READ1 genotypes and functional connectivity

Next, based on the seed region that showed significant associations between ICD parameters and the comprehension phenotype, we defined a resting state network. To do this, we constructed a map detailing the strength of the correlation between each voxel’s time course and the time course of the seed region (*3dDeconvolve*, followed by *3dcalc* to convert *R*^2^ values to *R* values). The *arctanh* transform was then applied to the data to convert correlation coefficients to z-scores (*3dcalc*). Network maps were then subjected to a groupwise *t*-test (*3dttest++*; *p* < 1 × 10^−20^).

Within this network, we identified peak nodes whose resting state time courses were highly correlated with the seed time course (*3dExtrema*; minimum separation distance of 30 mm, or 10 voxels). For each of these nodes, spheres were centered on peak co-ordinates (radius 6 mm, or two voxels; total volume 33.5 voxels each 3 × 3 × 3 mm in size), and the mean z-score from each subject-specific map was extracted for each sphere (*3dROIstats*). Then, for each node, we performed multiple regression analyses to assess the relationship between READ1 genotypes and the strength of the functional association between nodes in the network. To identify important covariates for these models, we performed backward selection using the R program ‘backward’ in the *mixlm* package^69^ with an inclusion/exclusion alpha criterion of .05 and the following initial model:

Connectivity ~ age + PIQ + sex + maternal education + SES + PC1 + PC2 + PC3

Once important covariates had been identified, we included these in final models as follows:

Connectivity ~ READ1 genotype + {important covariates identified from backward selection}.

## Data Availability

Anonymized data will be made available through collaborative agreement with the PI, Dr. Jeffrey R. Gruen, as specified in the informed consent for the GRaD Study. Interested potential collaborators are urged to contact Dr. Gruen at jeffrey.gruen@yale.edu.
